# Prevention of contrast induced nephropathy; a cardiology point of view

**Published:** 2015-01-01

**Authors:** Mohammad Hashemi, Mohaddeseh Behjati, Shiva Izadi

**Affiliations:** ^1^Departmentof Cardiology, Isfahan University of Medical Sciences, Isfahan, Iran; ^2^Isfahan Cardiovascular Research Institute, Isfahan University of Medical Sciences, Isfahan, Iran; ^3^Isfahan Heart Failure Research Institute, Isfahan University of Medical Sciences, Isfahan, Iran

**Keywords:** Contrast induced nephropathy, Renal failure, Contrast media

**Implication for health policy/practice/research/medical education:**

Contrast induced nephropathy (CIN) is one of the most common causes of hospital-acquired renal insufficiency. Among risk factors for susceptibility to CIN, the most commonly reported risk factors includes diabetes mellitus, hypotension, hypertension, pre-existing renal failure, anti-hypertensive agents, high total contrast volume and osmolality, advanced age and female gender.

By increasing the number of cardiovascular procedures, iodinated contrast media (ICM) is one of the most common agents used for diagnostic and prognostic and also therapeutic cardiac interventions. Contrast induced nephropathy (CIN) is one of the most leading causes of hospital-acquired renal insufficiency (1). Following cardiovascular invasive procedures, CIN occurs in about 3.1 to 31% of patients (2). CIN induces unfavorable outcomes and is associated with increased morbidity and mortality rate in hospitalized patients (3). This is a multifactorial disease, however, various pathophysiologic mechanisms have been attributed to development of CIN such as; direct toxicity to tubular cells, renal ischemia and hypoperfusion, oxidative stress, direct pro-apoptotic effects, altered vasomotor balance, enhanced vasoconstriction due to increased adenosine, endothelin, free radicals and decreased vasodilation due to diminished nitric oxide and prostaglandin levels (Figure 1) (4). Consequently, decreased renal perfusion and medullary ischemia contribute to the development and progression of CIN, but the real cause is still unknown (5,6). Hereby, we are going to introduce a memorial name for better identification of at risk patients derived from the risk factors with strongest odd’s ratio.

**Figure 1:**
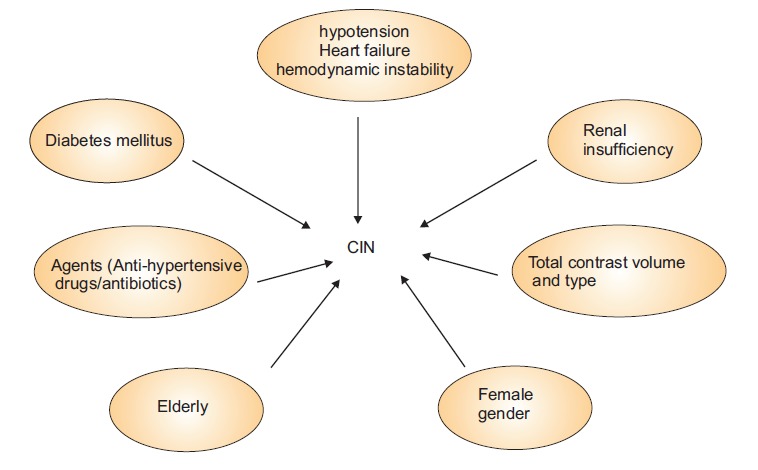
Pathophysiology of contrast induced nephropathy (CIN)

In a survey of full-text searches of electronic databases we have collected risk factors for CIN. Among them, the most common reported risk factors were focused. This would not be a scoring system but this acronym introduce an easy memorial name in order to estimate risk factors of CIN in cases undergoing coronary angiography and intervention to prevent occurrence of CIN.

Since the most commonly reported risk factors related to the occurrence of CIN were hypotension/hypertension/heart failure/hemodynamic instability, diabetes mellitus (DM), renal insufficiency, agents as antihypertensive drugs, antibiotics, non-steroidal anti-inflammatory drugs/anemia, total contrast volume and type, elderly, female gender; the memorial word could be hydrate-her (7-11).

Hereby, we introduce a memorial prediction system based on the predisposing risk factors with strongest odds ratio for development of CIN. Regarding hydrate-her, a brief explanation is provided in the following section. Hemodynamic instability extremely predisposes patients to the development of CIN as in the case of intra-aortic balloon use and sepsis (7,12). Pre-existing renal insufficiency has been defined as creatinine level of >1.2 mg/dl (13). Indeed, patients with glomerular filtration rate (GFR) level below 30 ml/min, 30-60 ml/min and >60 ml/min were considered as high risk patients, moderate risk and low risk, respectively (14). Hypotension was defined by some experts as systolic blood pressure <80 mmHg for at least 1 hour which requires inotropic support. High total contrast volume was suggested as total dose of contrast >200 ml (15). Indeed, the role of osmolality of contrast media has been well elucidated previously. Higher rate of CIN was seen using high osmolality contrast media (>1500 mOsm/kg) compared with low-osmolality (550-850 mOsm/kg) (16). Among anti-hypertensive agents, angiotensin converting enzyme inhibitors (ACEI) and renin-angiotensin blockers (RAS blockers) were most paid attention agents. Age more than 75 years old is considered as a potent risk factor for susceptibility to CIN occurrence (10). Avoidance of unnecessary hypoxia and hypotension, using minimum amount of contrast agent and withdrawal of nephrotoxic drugs seem to be the best preventive measures. Some therapeutic options as forced dieresis by adequate volume expansion, bicarbonate sodium, n-acetylcysteine and statins are advised (17,18). But since, current therapeutic strategies are not so effective for CIN, routine prediction and identification of at-risk cases, prevention of its occurrence and early detection are so beneficial. In the cases with any of above mentioned risk factors, hydration is the best practical approach for prevention of CIN. Indeed, the total contrast volume should be reduced to diminish the rate of CIN. Thus, physicians could predict cases at risk for development of CIN that could predict the occurrence of CIN by this memorial phrase.

Using this approach, cases with 0 score are at the lowest risk state. In cases that have a risk factor embedded in this memorial name should be hydrated well before the procedure.

## Conclusion

CIN is one of the most common causes of hospital-acquired renal insufficiency. Among risk factors for susceptibility to CIN, the most commonly reported risk factors includes diabetes mellitus, hypotension, hypertension, pre-existing renal failure, antihypertensive agents, high total contrast volume and osmolality, advanced age and female gender.

## Authors’ contributions

All authors contributed to the manuscript equally.

## Conflict of interests

The authors declared no competing interests.

## Ethical considerations

Ethical issues (including plagiarism, misconduct, data fabrication, falsification, double publication or submission, redundancy) have been completely observed by the authors.

## Funding/Support

None.
